# Health-related quality of life trajectories one year after COVID-19–induced ARDS: A secondary analysis of the CONFIDENT trial

**DOI:** 10.1016/j.aicoj.2025.100009

**Published:** 2026-01-16

**Authors:** Anne-Françoise Rousseau, Nadia Dardenne, Axelle Bertrand, Michael Piagnerelli, Eric Hoste, David Grimaldi, Isabelle Michaux, Elisabeth De Waele, Alexander Dumoulin, Philippe G Jorens, Emmanuel van der Hauwaert, Frédéric Vallot, Stoffel Lamote, Walter Swinnen, Nicolas De Schryver, Vincent Fraipont, Nathalie de Mey, Nicolas Dauby, Nathalie Layios, Jean-Baptiste Mesland, Anne-Françoise Donneau, Pierre-François Laterre, Benoit Misset

**Affiliations:** a Department of Intensive Care and Burn Center, University Hospital of Liège, Liège, Belgium; b Research Unit for a Life-Course Perspective on Health & Education-RUCHE, University of Liège, Liège, Belgium; c Biostatistics Center (B-STAT), University Hospital and University of Liège, Liège, Belgium; d Department of Intensive Care Medicine, Centre Hospitalier Universitaire (CHU) de Charleroi–Marie Curie Hospital, Université Libre de Bruxelles, Charleroi, Belgium; e Department of Intensive Care Medicine, Ghent University Hospital, Ghent, Belgium; f Department of Intensive Care Medicine, Cliniques Universitaires de Bruxelles–Erasme, Free University of Brussels, Brussels, Belgium; g Department of Intensive Care, Université Catholique de Louvain (UCL), CHU UCL Namur, Yvoir, Belgium; h Department of Intensive Care Medicine, Brussels University Hospital, Vrije Universiteit Brussel, Jette, Belgium; i Department of Intensive Care Medicine, Delta General Hospital, Roeselare, Belgium; j Department of Intensive Care Medicine, Antwerp University Hospital, University of Antwerp, Lemp, Edegem, Belgium; k Department of Intensive Care Medicine, Imelda General Hospital, Bonheiden, Belgium; l Department of Intensive Care Medicine, Wallonie Picarde General Hospital, Tournai, Belgium; m Department of Intensive Care Medicine, Groeninge General Hospital, Kortrijk, Belgium; n Department of Intensive Care Medicine, Sint Blasius General Hospital, Dendermonde, Belgium; o Department of Intensive Care Medicine, Saint-Pierre Medical Clinic, Ottignies, Belgium; p Department of Intensive Care Medicine, Citadelle General Hospital, Liège, Belgium; q Department of Intensive Care Medicine, Onze-Lieve-Vrouw General Hospital, Belgium; r Infectious Diseases, Saint-Pierre University Hospital, Brussels, Belgium; s Department of Intensive Care Medicine, Saint-Luc University Hospital, Brussels, Belgium; t Department of Intensive Care Medicine, Centre Hospitalier Régional Mons-Hainaut, Mons, Belgium

**Keywords:** COVID-19, Critical care, Critical illness, Survivors, Health-Related quality of life

## Abstract

**Rationale:**

Survivors of ARDS are at risk of persistent physical and psychological impairments, yet reliable prognostic factors for long-term recovery are poorly defined. The aims of the study were to describe changes in health-related quality of life (HRQoL) during the year after discharge from intensive care unit (ICU) in a cohort of ARDS survivors, and to identify factors associated with a favorable recovery trajectory.

**Methods:**

This planned secondary analysis used prospectively collected data from the multicenter randomized CONFIDENT trial that enrolled 475 mechanically ventilated COVID-19 ARDS patients. Patients who completed interviews at both day 90 (D90) and one year (Y1) were included. HRQoL was assessed using the EQ-5D-5L utility score (EQ-score) and visual analog scale (EQ-VAS). Baseline status, disease severity, and ICU characteristics were analyzed for associations with HRQoL changes.

**Results:**

156 survivors completed follow-up at both D90 and Y1. EQ-score and EQ-VAS significantly improved between D90 and Y1 (p < 0.0001 and p = 0.0002 respectively), but both remained lower than pre-ICU status. Notably, 38 and 43% of patients showed stagnation or deterioration in EQ-score and EQ-VAS over the year. Longer durations of mechanical ventilation, ICU stay, and hospital stay were associated with greater EQ-score and EQ-VAS recovery, whereas shorter stays were linked to less improvement (respectively p = 0.0002 and p = 0.025, p = 0.0002 and p = 0.0035, p = 0.0020 and p = 0.026). Demographics and pre-admission frailty showed no impact on the recovery trajectory.

**Conclusion:**

In this multicenter cohort of ARDS survivors, patients with shorter durations of mechanical ventilation, ICU and hospital stay experienced poorer HRQoL recovery, independently of baseline characteristics such as age or frailty.

**Trial registration:**

Clinicaltrials.gov registration number NCT04558476. Registered 14 September 2020—Retrospectively registered, https://clinicaltrials.gov/ct2/show/NCT04558476

## Introduction

The acute respiratory distress syndrome (ARDS) is a one of the most severe complications of sepsis from pulmonary or systemic origin. Between 2020 and 2022, coronavirus disease 2019 (COVID-19) emerged as the leading cause of ARDS. In the CONFIDENT trial, a multicenter, randomized, controlled study conducted in mechanically ventilated patients with COVID-19-induced ARDS, convalescent plasma was shown to improve day-28 survival [1]. This trial also prospectively included long-term outcomes as pre-planned secondary endpoints, assessed at day 90 (D90) and one year (Y1) after discharge of the intensive care unit (ICU). Alongside the pandemic, ARDS remains highly prevalent and frequently requires prolonged mechanical ventilation [2]. Surviving ARDS is associated with significant long-term sequelae, even in young patients without significant underlying illnesses [3,4].

Post-intensive care syndrome (PICS) is a general term referring to a combination of new or worsening disorders in physical (neuromuscular weakness and reduced autonomy for activities of daily living), mental, neurocognitive and additional metabolic or functional domains [5,6]. PICS may persist for months to years, and negatively impacts capacity to regain independence, quality of life, and healthcare related costs [7]. PICS and its consequences can be detected by a number of clinical parameters or patient-reported outcomes measures (PROM), depending on the considered domain [8]. Health-related quality of life (HRQoL) is recognized as highly meaningful to patients [9].

Post-ICU follow-up clinics have been increasingly advocated as a means to detect and address PICS. However, their implementation remains limited due to resource constraints, unclear benefit, and the absence of standardized patient selection criteria [10]. Predicting which patients will experience long-term recovery or persistent impairments remains challenging, as markers used during the acute phase may not accurately reflect post-discharge trajectories [11]. Identifying such predictors could help refine selection criteria for post-ICU care pathways.

Against this backdrop, the CONFIDENT cohort offers a unique opportunity to study HRQoL recovery in a well-defined, homogeneous population of ARDS survivors. The aims of the study were to describe changes in HRQoL during the year after ICU discharge in a cohort of COVID-19 induced ARDS survivors, and to identify factors associated with a favorable recovery trajectory.

## Methods

### Participants - data sources

Patients were identified from the previously published CONFIDENT trial [1]. This study was conducted according to the guidelines of the Declaration of Helsinki (1964) and its later amendments. It was approved by the Central Ethics Committee of the University hospital of Liège, Belgium on September 1, 2020, number # 2020/239.

Briefly, the CONFIDENT study was a multicenter Belgian randomized open-label trial evaluating the use of convalescent plasma with a high neutralizing antibody titer on death by day 28 in patients with COVID-19-induced ARDS and mechanically ventilated for less than 5 days. Patients were included if they also fulfilled the following criteria: Clinical Frailty Scale (CFS) <6 before hospitalization, absence of a prior episode of transfusion-related side effects, and absence of medical decision to limit therapy. Besides the administration of 2 units (400−500 mL in total) of convalescent plasma once within the 24 h of inclusion in the treated group (CP group), all other clinical decisions were left to the discretion of the ICU teams, according to standards of care (SOC group). The CONFIDENT study was conducted during the second and third Belgian waves of the COVID-19 pandemic. Wave 2 corresponded to the period from October 1 st to November 30th, 2020, while wave 3 encompassed the period after November 30th, 2020. All participating centers were operating under the national surge-response framework, which ensured standardized clinical management and inter-hospital coordination during periods of increased ICU pressure.

Data collected included patients demographics and characteristics (age, sex, body mass index / BMI, comorbidities, alcohol and smoking status, educational level, employment status), baseline (pre-ICU) HRQoL before admission (described by relatives), disease characteristics (score on the Acute Physiology and Chronic Health Evaluation / APACHE II at admission (D0), Sequential Organ Failure Assessment / SOFA score and C-reactive protein / CRP level in blood, both at D0 and day 7 (D7)), ICU and hospital characteristics (duration of mechanical ventilation, need for renal replacement therapy and extracorporeal membrane oxygenation, ICU and hospital length of stay).

Other data collected (as secondary outcomes of the CONFIDENT trial) included mental health and HRQoL at D90 and Y1. These data were collected by phone by a trained clinical research assistant dedicated to this task by the coordinating center. Patients who filled out the remote at least one assessment of quality of life at D90 and Y1were included in the present secondary analysis.

Post-ICU mental health status was assessed using the Hospital Anxiety and Depression scale (HADS). The HADS consists of two 7-item subscales evaluating symptoms of depression (seven items – HADS-D subscale) and symptoms of anxiety (seven items – HADS-A subscale) [12]. The standard cutoff threshold value of >7 out of 21 on either subscale was used to define a borderline status (8–10) or clinically significant status (11–21) of depression or anxiety, respectively.

### HRQoL outcome

Health-related quality of life (HRQoL) was measured using the EQ-5D-5L (Supplemental Fig. S1). This tool comprises two sections. The first section includes a five-question descriptive component exploring five dimensions: mobility, self-care, usual activities, pain/discomfort and anxiety/depression. Each dimension is scored on a five-point scale: 1, no problem; 2, slight problem; 3, moderate problem; 4, severe problem; and 5, unable to do/extreme problem. The 5L responses were converted to a utility score (value) using recently published preference-based scoring systems derived from the general Belgian population (EQ-score) [13]. The second section is a visual analogue scale (EQ-VAS) measuring patient-perceived overall health from 0 to 100, where 0 is the worst imaginable health status, while 100 is the best.

### Statistical analyses

Statistical analysis was carried out using SAS version 9.4. Qualitative variables were expressed as counts and percentages. Normality of the distribution of the quantitative variables was investigated graphically with histograms and quantile-quantile plots and assessed using the Shapiro–Wilk hypothesis test. As quantitative parameters were not all normally distributed, results were expressed as median (P50) and interquartile range (P25-P75). Missing values were not replaced. The nonparametric Mann–Whitney test, the chi-squared test or the Fisher exact test were used for intergroups comparisons, as appropriate. Changes in quality of life were assessed using the non-parametric Wilcoxon signed-ranks test. Patients were grouped according to their HRQoL evolution: Group Y1 > D90, Group Y1 = D90 and Group Y1 < 90, for those who experienced an improvement, a status quo or a worsening of their quality of life at Y1 compared to D90, respectively. Group Y1 = 90 and Group Y1 < 90 were gathered in a unique Group Y1 ≤ D90 to assess the association between HRQoL evolution and patients’characteristics. The level of uncertainty was set at 5%.

## Results

### Patients

**Fig. 1:**
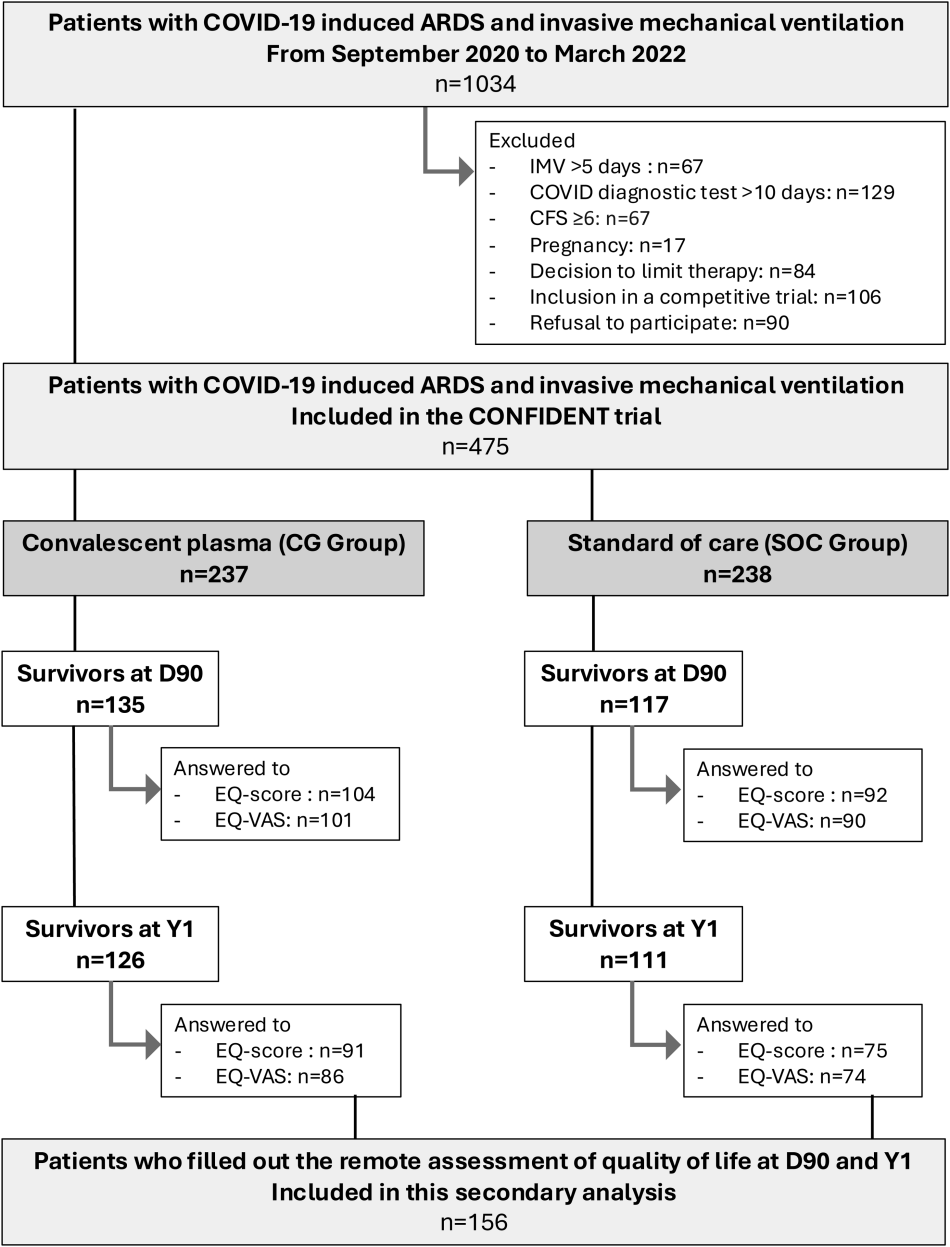
Flow chart.

Of a total of 475 COVID-19 patients included in the CONFIDENT trial from September 2020 through March 2022, 156 survived and completed the remote assessment at D90 and Y1 and were therefore included in this study (Fig. 1). Their characteristics are described in Table 1.

**Table 1:** Baseline, ICU and post-ICU characteristics of patients included in the present analysis (n = 156) and of patients from the CONFIDENT trial who survived at Y1 but were not included in the present analysis (n = 81).

Characteristic		Patients included in CONFIDENT trial, who survived at Y1, and who were included in the present analysis		Patients included in CONFIDENT trial, who survived at Y1, but who were not included in the present analysis		p value
		n	Values	n	Values	
Age, years		156	60.5 (51.5−68)	81	59 (47−65)	0.35
Sex (female), n (%)		156	60 (38.5)	81	22 (27.2)	0.083
BMI at D0, kg/m^2^		141	31 (27.4–35.4)	76	31.8 (27.6−35.5)	0.53
Educational level, n (%)	Secondary education	83	58 (69.9)	54	32 (59.3)	0.17
	Bachelor’s degree		12 (14.5)		12 (22.2)	
	Master’s degree		5 (6)		2 (3.7)	
	Doctorate		3 (3.6)		0 (0)	
	Other		5 (6)		8 (14.8)	
Employment status, n (%)	Employed	108	49 (45.4)	60	25 (41.7)	0.018
	Unemployed		9 (8.3)		6 (10)	
	Retired		42 (38.9)		23 (38.3)	
	Sick leave		7 (6.5)		0 (0)	
	Other		1 (0.9)		6 (10)	
Clinical Frailty Scale		156	3 (2−3)	81	2 (1−3)	0.21
Comorbidities, n (%)	Diabetes	156	48 (30.8)	80	28 (35)	0.51
	Active smoking	141	10 (7.1)	76	4 (5.3)	0.77
	Active alcohol	141	6 (4.3)	74	3 (4.1)	1
	Long-term steroids	156	24 (15.4)	81	6 (7.4)	0.08
	Immunotherapy	156	7 (4.5)	81	1 (1.2)	0.27
	Chemotherapy	156	1 (0.6)	81	2 (2.5)	0.27
APACHE II		156	12 (9−15)	81	9 (7−12)	0.0002
D0	SOFA	156	6 (4−7)	81	4 (3−6)	0.025
	CRP, mg/L	156	119.2 (64.3−182.5)	81	98 (55−160.5)	0.15
D7	SOFA	152	4 (3−6)	73	4 (3−5)	0.043
	CRP, mg/L	144	83.7 (27−157.2)	80	66 (27−137.5)	0.26
Mechanical ventilation duration, days		155	17 (10−27)	81	15 (9−24)	0.46
Renal replacement therapy, n (%)		156	12 (7.7)	81	1 (1.2)	0.039
ECMO, n (%)		156	24 (15.4)	81	13 (16)	0.89
ICU LOS, days		156	26 (14.5−46)	81	21 (13−36)	0.031
Hospital LOS, days		156	51 (27−78)	81	27 (21−49)	<0.0001
Hospital discharge location, n (%)	Home	155	100 (65.2)	81	44 (54.3)	0.26
	Rehabilitation site	155	45 (29)	81	32 (39.5)	
	Long-term residency	155	1 (0.6)	81	0	
	Other hospital	155	7 (4.6)	81	3 (3.7)	
	Other	155	1 (0.6)	81	2 (2.5)	
D90	HADS-Anxiety	133	4 (1−7)	42	2 (0−10)	0.72
	HADS-Depression	136	3 (1−7)	42	2.5 (1−5)	0.55
Y1	HADS-Anxiety	133	4 (1−8)	12	6.5 (2.5−9.5)	0.27
	HADS-Depression	136	3 (1−7)	12	6.5 (1−8)	0.20

Data are expressed as n (%) or median (P25-P50).

APACHE: Acute Physiology And Chronic Health Evaluation; BMI: body mass index; D: day; CRP: C-reactive protein; ECMO: Extracorporeal Membrane Oxygenation; HADS: Hospital Anxiety and Depression scale; ICU: intensive care unit; LOS: length of stay; SOFA: Sepsis-related Organ Failure Assessment; Y: year.

Compared to the 81 patients who survived at Y1 but who did not complete the remote assessment at D90 and Y1, the included patients had a more severe illness that was associated with a longer ICU and hospital stay (Table 1).

The ICU severity scores, length of ICU stay, and discharge destinations were found to be very similar between the two pandemic waves (Supplemental Table S1).

HADS scores at D90 and Y1 are described in Table 1. The proportion of included patients presenting signs of anxiety and depression (HADS subscores ≥11) was respectively 20/156 (15%) and 12/156 (9%) at D90 and 9/156 (6.6%) and 14 (10.3%) at Y1. No significant change in HADS-A and HADS-D was observed between D90 and Y1 (respectively p = 0.62 and p = 0.82).

### Health-related quality of life at D90 and Y1

The evolution of EQ-score and EQ-VAS at D90 and Y1, compared to pre-admission status, are presented in Table 2 for the studied cohort and for the two groups of treatment (CP and SOC groups). The evolution of the five dimensions of the EQ-5D-5L in the studied cohort is detailed in the Supplemental Table S2. EQ-score and EQ-VAS did not differ between CP and SOC groups at any timepoints. Both PROMs were significantly lower at D90 and Y1 compared to pre-ICU status and significantly increased at Y1 compared to D90.

**Table 2:** Pre-ICU, D90 and Y1 outcomes in the studied population and in the two groups of treatment (CP and SOC groups).

Outcomes		Studied population (n = 156)		CP group (n = 86)		SOC group (n = 70)		p value (comparison CP group vs SOC group)
		n	values	n	values	n	values	
EQ-score	Pre-ICU	79	0.90 (0.78−1)	43	0.90 (0.73−1)	36	1 (0.84−1)	0.11
	D90	148	0.78 (0.51−0.90)	79	0.76 (0.45−0.90)	69	0.79 (0.60−0.90)	0.46
	Y1	151	0.84 (0.68−0.93)	83	0.83 (0.67−0.94)	68	0.86 (0.73−0.93)	0.43
	Difference Y1-D90	144	0.07 (−0.02−0.25)	77	0.09 (−0.01−0.26)	67	0.06 (−0.02−0.24)	0.89
*p value D90 vs pre*		*<0.0001*		*0.0028*		*0.0014*		
*p value Y1 vs pre*		*0.0087*		*0.14*		*0.014*		
*P value Y1 vs D90*		*<0.0001*		*0.0003*		*0.0008*		
EQ-VAS	Pre-ICU	71	80 (70−90)	36	75 (65−82.5)	35	80 (70−90)	0.068
	D90	144	70 (57.5−77.5)	76	70 (57.5−75)	68	70 (57.5−80)	0.81
	Y1	145	70 (65−85)	78	75 (60−85)	67	70 (65−85)	0.99
	Difference Y1-D90	135	5 (−5−15)	70	5 (−5−15)	65	5 (0−20)	0.67
*p value D90 vs pre*		*0.0001*		*0.046*		*0.0005*		
*p value Y1 vs pre*		*0.017*		*0.28*		*0.028*		
*P value Y1 vs D90*		*0.0002*		*0.014*		*0.0054*		

Data are expressed as median (P25-P75).

CP: convalescent plasma; D: day; EQ: EuroQOL; SOC: standard of care; VAS: visual analogue scale; Y: year.

**Fig. 2:**
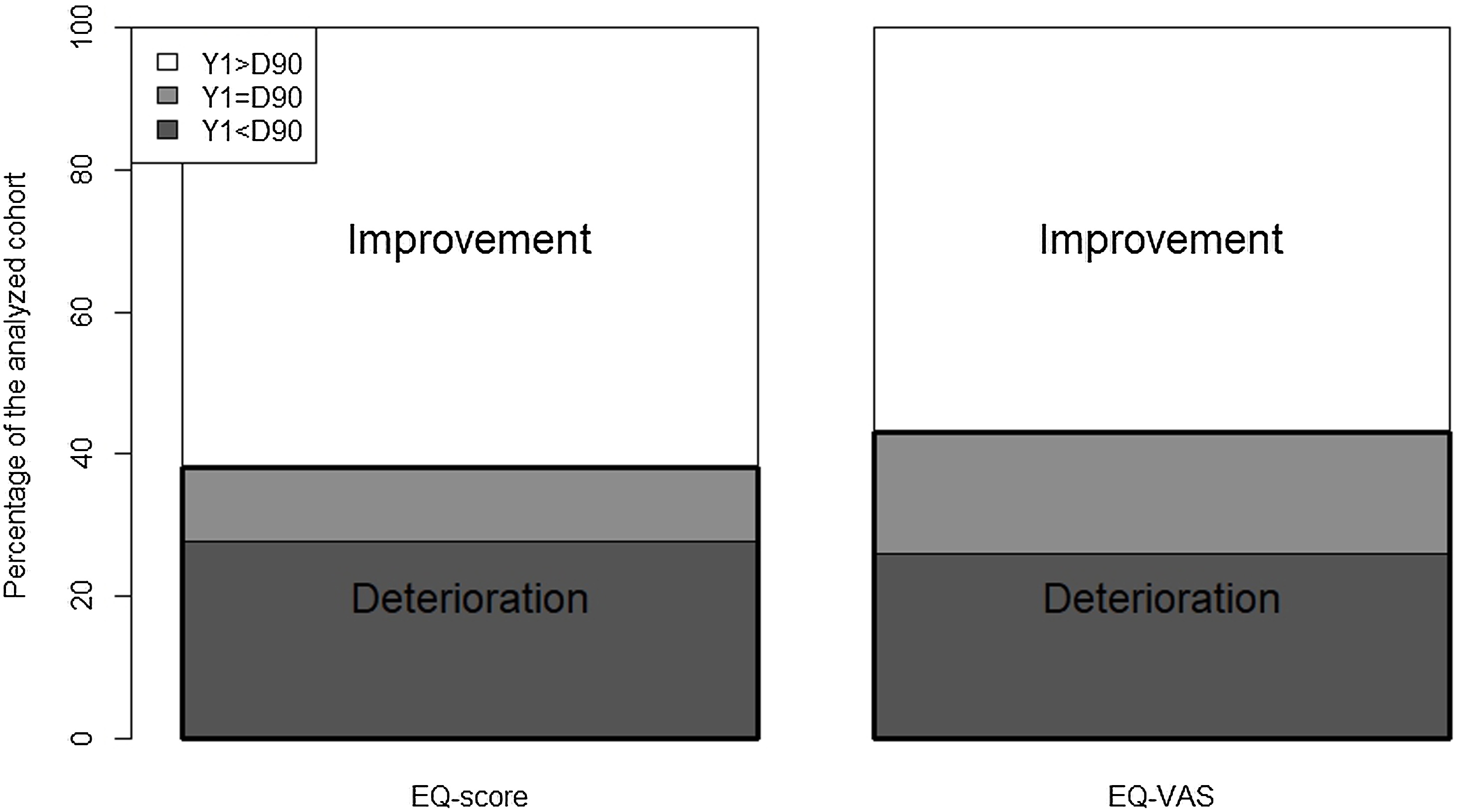
Proportions of patients experiencing an improvement, a status quo or a deterioration of their HRQoL (EQ-score and EQ-VAS) between D90 and Y1. HRQoL improved in 62% and 57% patients, based on the EQ-score and the EQ-VAS, respectively. D: day; EQ: EuroQOL; HRQoL: health-related quality of life; VAS: visual analogue scale; Y: year.

The proportions of patients who experienced an improvement, a status quo or a worsening of their quality of life at Y1 compared to D90 are depicted in Fig. 2. HRQoL improved in 57% and 62% patients, based on the EQ-VAS and the EQ-score, respectively. The description of the EQ-score and EQ-VAS values at each timepoint in the three subgroups is detailed in Table 3.

**Table 3:** Health-related quality of life at D90 and Y1 in three subgroups defined according to the evolution of HRQoL during the year of follow-up (Group Y1 > D90, Group Y1 = D90 and Group Y1 < 90, for those who experienced an improvement, a status quo or a worsening of their quality of life at Y1 compared to D90, respectively).

Outcomes		Group Y1 > D90		Group Y1 = D90		Group Y1 < D90	
		n	values	n	values	n	values
EQ-score	D90	89	0.65 (0.32−0.81)	15	1 (0.94−1)	40	0.90 (0.78−0.93)
	Y1	89	0.84 (0.72−0.93)	15	1 (0.94−1)	40	0.75 (0.58−0.84)
EQ-VAS	D90	77	60 (50−70)	23	70 (70−80)	35	80 (70−85)
	Y1	77	75 (70−85)	23	70 (70−80)	35	60 (50−70)

Data are expressed as median (P25-P75).

D: day; EQ: EuroQOL; HRQoL: health-related quality of life; VAS: visual analogue scale; Y: year.

### Risk factors for health-related quality of life alterations after ICU

**Fig. 3:**
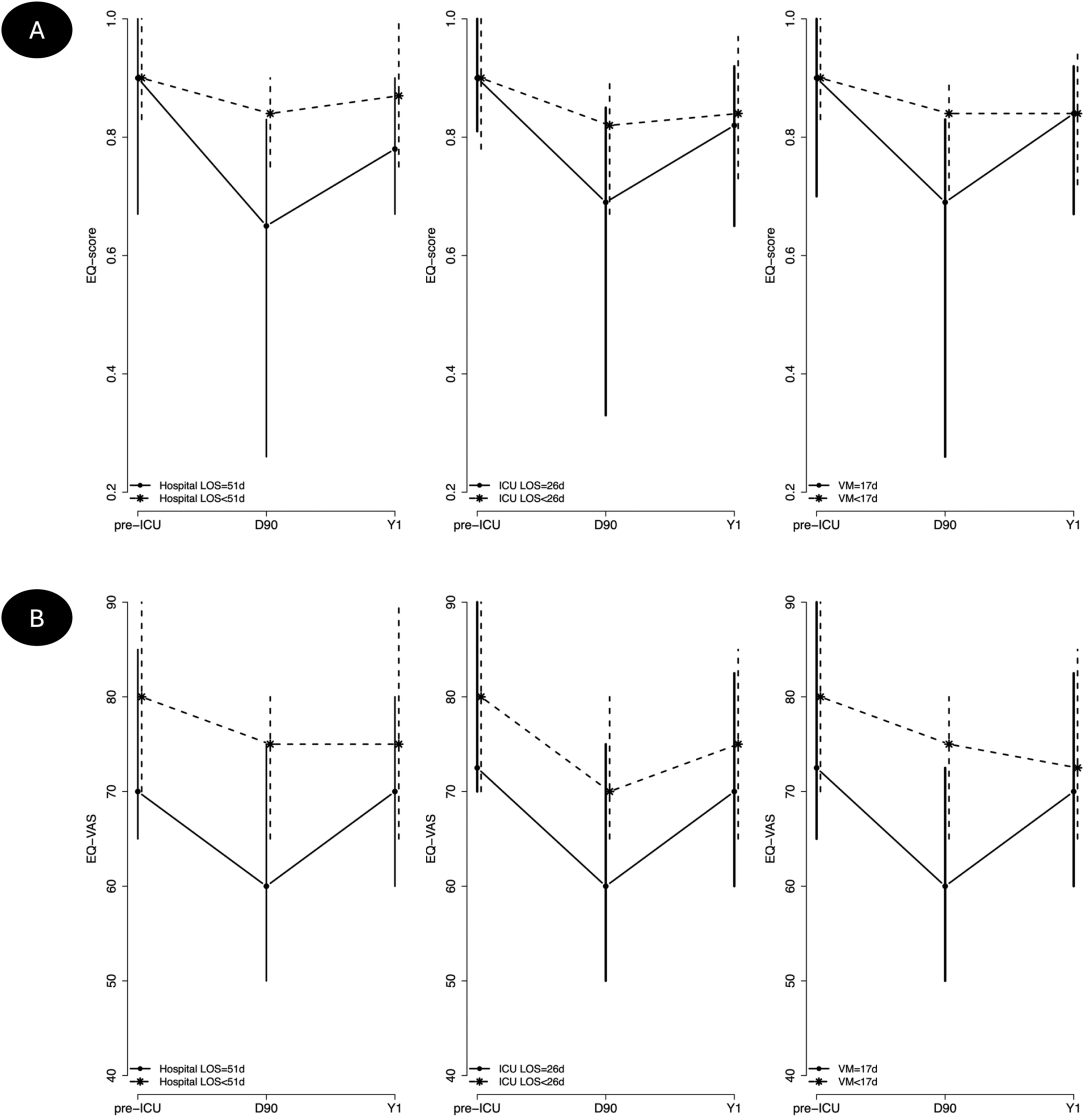
Evolution of the EQ-score (A) and EQ-VAS (B) between D90 and Y1in patients with MV duration, ICU LOS and hospital LOS above or below their respective medians. Values were lower at both D90 and Y1 in sicker patients, but their progression was better from D90 to Y1. A substantial variability in post-ICU trajectory is observed among the cohort. D: day; EQ: EuroQOL; ICU: intensive care unit; LOS: length of stay; MV: mechanical ventilation; VAS: visual analogue scale; Y: year. Data are represented as median (P25-P50).

Association between EQ-score and EQ-VAS evolution and clinical and biological characteristics are shown in Supplemental Tables S3 and S4, respectively. Improvement in both EQ-score and EQ-VAS between D90 and Y1 was significantly associated with longer durations of mechanical ventilation (p = 0.0002 and p = 0.025), ICU stay (p = 0.0002 and p = 0.0035) and hospital stay (p = 0.0020 and p = 0.026). These three parameters were highly correlated, precluding any inclusion in a multivariate model. Improvement in EQ-score was also associated with higher educational levels (p = 0.047), while improvement in EQ-VAS was also associated with male gender (p = 0.043). The Fig. 3 depicts the evolution of EQ-score and EQ-VAS between D90 and Y1 in patients with duration of mechanical ventilation, ICU stay and hospital stay above or below their respective medians. Consistently, values were lower at both D90 and Y1 in sicker patients, but their progression was better from D90 to Y1.

Among the cohort of patients included in this secondary analysis, 37/156 (23.7%) had not recovered their baseline EQ-score and EQ-VAS scores at Y1. Within this subgroup, those who showed improvement between D90 and Y1 continued to exhibit significantly longer ICU length of stay and longer duration of mechanical ventilation compared with those who did not improve (see Supplemental Table S5).

## Discussion

In this multicenter cohort of mechanically ventilated COVID-19 ARDS survivors from the CONFIDENT trial, almost a quarter of the patients did not recover their baseline HRQoL at Y1. Approximately 38% and 43% of patients experienced no improvement—or even deterioration—in HRQoL one year after ICU discharge, based on the EQ-score and the EQ-VAS respectively. Greater improvements in HRQoL were associated with longer durations of mechanical ventilation, ICU stay, and hospital stay, while patients with shorter ICU exposure showed more limited HRQoL recovery. These observations were independent of baseline characteristics such as age or frailty.

There is a widespread assumption that sicker patients have poorer long-term outcomes [7]. The present paradoxical results challenge the assumption that resilience is greater in patients with short ICU stays, suggesting that these patients may be overlooked in post-ICU care due to under-recognized vulnerability. Our findings align with recent studies showing that PICS can affect patients regardless of acute illness severity or ICU length of stay [14,15]. So far, traditional indicators—such as ICU length of stay—are often used as surrogate markers to allocate follow-up resources [16,17], yet our data suggest they may be insufficient. Functional status at ICU discharge — encompassing physical, psychological, cognitive, social, and metabolic domains — and early post-discharge Patient-Reported Experience Measures (PREMs) could be explored in future studies to determine whether their addition to traditional parameters such as ICU length of stay improves the prediction of recovery capacity [[18], [19], [20], [21]].

Despite cohort homogeneity in terms of initial pathology and ICU management, we observed substantial variability in outcomes and post-ICU trajectories. This variability, increasingly reported in the recent studies [22], likely reflects unmeasured factors—including complex psychological, social, and rehabilitative dimensions—that are rarely captured in clinical trials [23]. The psychological parameters assessed in this study were limited to anxiety and depression, measured through the Hospital Anxiety and Depression Scale (HADS), which provides only a coarse screening of these domains. Nevertheless, this study confirms the prevalence of anxiety and depression symptoms in COVID-19 ARDS survivors, and the stability of the psychological burden between D90 and Y1, as also observed recently in patients who survived ARDS of different infectious origin [24]. From a clinical perspective, our findings reinforce the importance of early screening for PICS and for a more personalized approach to post-ICU care. Rather than applying a one-size-fits-all strategy, follow-up trajectories should be tailored to each patient's evolving profile, vulnerabilities, and potential for recovery.

Although the pandemic context was unique—particularly with regard to uniformly restrictive family-visitation policies that may have influenced psychological outcomes and patients’ perceived quality of life [25] —existing evidence indicates that COVID-19 and non-COVID survivors tend to exhibit broadly similar long-term outcomes at both 6 months [26] and 1 year [27] after discharge. On this basis, while caution is needed when extrapolating beyond the COVID-19 population, it is not unreasonable to consider that some of the patterns observed here may apply to other critically ill populations. Nonetheless, ICU populations are inherently heterogeneous, with recovery trajectories that can differ substantially depending on the underlying pathology leading to admission [28]. This heterogeneity justifies a mitigated interpretation of the generalizability of our findings.

This study offers a longitudinal perspective on HRQoL in a highly defined cohort of ARDS survivors and raises important clinical and conceptual questions about how we assess and anticipate recovery after critical illness. The homogeneity of this cohort reduced the confounding effects inherent to mixed ICU cohorts. However, the homogeneity of the cohort was partly due to the inclusion and exclusion criteria of the CONFIDENT trial, selecting people without extreme frailty and without therapy limitation. It is possible that such selection produced better outcomes than those observed in unselected ICU population. reflect the full spectrum of unselected ICU patients, whose trajectories vary widely depending on underlying pathologies, comorbidities, and baseline vulnerabilities. This restricts the generalizability of our findings to broader ICU populations. Moreover, CONFIDENT patients included in the present analysis had higher APACHE II and SOFA scores compared with those who were not, which could reflect another selection bias toward more severe cases. It is also not excluded that the homogeneity of the cohort could have limited the demonstration of predictive factors of recovery, not identifying subgroups with alternative background or clinical patterns. Some limitations should be noted. First, despite prospective data collection, only 156 of the 475 CONFIDENT patients were analyzable at both D90 and Y1, introducing potential attrition bias. Comparison with non-respondents suggested that those included had more severe acute illness. This may have led to an underestimation of the burden in milder cases or, conversely, to a selection of more engaged survivors. Second, pre-ICU status was described by relatives (as patients were unresponsive) and was not confirmed a posteriori by patients themselves. Even if inherent to the protocol and to the clinical conditions, this could have biased the longitudinal analysis. Third, the absence of detailed data on post-ICU rehabilitation and post-ICI follow-up prevents us from distinguishing spontaneous versus intervention-driven recovery. Lastly, the limited number of predictors explored reflects the original trial design, not focused primarily on long-term recovery.

## Conclusion

In this multicenter cohort of COVID-19 ARDS survivors who benefited from mechanical ventilation during the ICU stay, quality of life remained substantially impaired one year after ICU discharge for a significant proportion of patients, despite some overall improvement over time. Patients with shorter durations of mechanical ventilation, ICU stay, and hospital stay appeared to experience more limited HRQoL recovery, independently of baseline characteristics such as age or frailty; however, these associations should be interpreted with caution given the specific characteristics of the CONFIDENT cohort and protocol. However, these findings challenge the assumption that traditional markers of acute severity, such as ICU length of stay, can reliably predict long-term outcomes and can be used as criteria to guide follow-up pathways. Our results highlight the need for early identification of patients at risk of persistent impairments, including those who may appear less severely ill during their ICU stay.

## CRediT authorship contribution statement

AFR, ND, MP, EH, AFD, PFL, BM designed research; AB, MP, EH, DG, IM, EDW, AD, PGJ, EVDH, FV, SL, WS, NDS, VF, NDM, ND, NL, JBM, PFL conducted research; AFR, ND, AFD, BM analyzed data; AFR wrote paper; ND, MP, IM, PGJ, NDS, VF, ND, NL, AFD, BM critically reviewed paper. All authors approved the final manuscript.

## Consent for publication

Not applicable.

## Ethical approval

This study was approved by the Central Ethics Committee of the University hospital of Liège, Belgium on September 1, 2020, number # 2020/239.

## Funding

Funded by the Belgian Health Care Knowledge Center.

## Previous presentation

Presented in part at the annual meetings of the French Intensive Care Society in Paris on June 13, 2024 and the European Society of Intensive Care Medicine in Barcelona, Spain on October 8, 2024.

## Availability of data and material

The datasets used and/or analyzed during the current study are available from the corresponding author on reasonable request.

## Declaration of competing interest

All authors declare that they have no competing interests.
