# Supplementary material for: Health-related quality of life trajectories one year after COVID-19–induced ARDS: A secondary analysis of the CONFIDENT trial
Source: Ann Intensive Care. 2026 Jan 16;16:100009. doi: 10.1016/j.aicoj.2025.100009 (PMC12934435; doi:10.1016/j.aicoj.2025.100009)
Supplement: Supplementary file 1 [file mmc1.docx]

**Health-related quality of life trajectories one year after COVID-19–induced ARDS: a secondary analysis of the CONFIDENT trial.**

**METHODS:**

**Supplemental Figure 1: Description of the EQ-5D-5L (including the EQ-score (A) and EQ-VAS (B))**

***Source: https://euroqol.org/information-and-support/euroqol-instruments/eq-5d-5l/***

**RESULTS:**

**Supplemental Table 1: Baseline, ICU and post-ICU characteristics of patients included in the present analysis (n=156), according to the Belgian pandemic waves (wave 2: from October 1^st^, 2020 to November 30^th^, 2020; wave 3: after November 30^th^, 2020)**

| **Characteristic** | | | **Wave 2** | | **Wave 3** | | **p value** |
| --- | --- | --- | --- | --- | --- | --- | --- |
|  |  |  | **n** | **Values** | **n** | **Values** |  |
| Age, years | | | 46 | 64.5 (57-69) | 110 | 58 (50-65) | 0.0015 |
| Sex (female), n (%) | | | 46 | 15 (32.6) | 110 | 45 (40.9) | 0.33 |
| BMI at D0, kg/m^2^ | | | 40 | 31.2 (26.9-34.5) | 101 | 30.9 (26.6-36.1) | 0.48 |
| Educational level, n (%) | Secondary education | | 25 | 15 (60) | 58 | 43 (74.1) | 0.028 |
|  | Bachelor’s degree | |  | 4 (16) |  | 8 (13.8) |  |
|  | Master’s degree | |  | 0 (0) |  | 5 (8.6) |  |
|  | Doctorate | |  | 2 (8) |  | 1 (1.7) |  |
|  | Other | |  | 4 (16) |  | 1 (1.7) |  |
| Employment status, n (%) | Employed | | 32 | 13 (40.6) | 76 | 36 (47.4) | 0.063 |
|  | Unemployed | |  | 5 (15.6) |  | 4 (5.3) |  |
|  | Retired | |  | 13 (40.6) |  | 29 (38.2) |  |
|  | Sick leave | |  | 0 (0) |  | 7 (9.2) |  |
|  | Other | |  | 1 (3.1) |  | 0 (0) |  |
| Clinical Frailty Scale | | | 46 | 3 (2-3) | 110 | 3 (2-3) | 0.83 |
| Pre-ICU HRQoL | | EQ-score | 23 | 0.9 (0.84-1) | 56 | 0.9 (0.77-1) | 0.91 |
|  |  | EQ-VAS | 19 | 80 (70-90) | 52 | 75 (66-90) | 0.38 |
| Comorbidities, n (%) | Diabetes | | 46 | 14 (30.4) | 110 | 34 (30.9) | 0.95 |
|  | Active smoking | | 44 | 5 (11.4) | 97 | 5 (5.2) | 0.29 |
|  | Active alcohol | | 43 | 2 (4.7) | 98 | 4 (4.1) | 1.00 |
|  | Long-term steroids | | 46 | 7 (15.2) | 110 | 17 (15.5) | 0.97 |
|  | Immunotherapy | | 46 | 0 (0) | 110 | 7 (6.4) | 0.11 |
|  | Chemotherapy | | 46 | 1 (2.2) | 110 | 0 (0) | 0.29 |
| APACHE II | | | 46 | 12 (8-17) | 110 | 12 (9-15) | 0.70 |
| D0 | SOFA | | 46 | 6 (4-8) | 110 | 5 (4-7) | 0.11 |
|  | CRP, mg/L | | 46 | 101.2 (52-198.3) | 110 | 122.2 (73-180.6) | 0.60 |
| D7 | SOFA | | 44 | 5 (3-7) | 108 | 4 (3-6) | 0.47 |
|  | CRP, mg/L | | 44 | 103.7 (30-155.4) | 110 | 81.1(25.4-167.2) | 0.77 |
| Mechanical ventilation duration, days | | | 46 | 13 (9-26) | 109 | 18 (10-29) | 0.15 |
| Renal replacement therapy, n (%) | | | 46 | 3 (6.5) | 110 | 9 (8.2) | 1.00 |
| ECMO, n (%) | | | 46 | 3 (6.5) | 110 | 21 (19.1) | 0.047 |
| ICU LOS, days | | | 46 | 21 (13-46) | 110 | 27 (15-46) | 0.26 |
| Hospital LOS, days | | | 46 | 35.7 (26-70) | 109 | 56 (28-88) | 0.16 |
| Hospital discharge location, n (%) | | Home | 46 | 25 (54.4) | 109 | 76 (69.7) | 0.089 |
|  |  | Rehabilitation site |  | 16 (34.8) |  | 29 (26.6) |  |
|  |  | Long-term residency |  | 1 (2.2) |  | 0 (0) |  |
|  |  | Other hospital |  | 4 (8.7) |  | 3 (2.8) |  |
|  |  | Other |  | 0 (0) |  | 1 (0.9) |  |
| D90 | | EQ-score | 45 | 0.76 (0.63-0.9) | 103 | 0.78 (0.46-0.9) | 0.98 |
|  |  | EQ-VAS | 43 | 70 (50-75) | 101 | 70 (60-80) | 0.54 |
|  |  | HADS-Anxiety | 39 | 6 (3-11) | 94 | 3 (0-7) | 0.012 |
|  |  | HADS-Depression | 39 | 3 (1-7) | 94 | 2.5 (1-7) | 0.87 |
| Y1 | | EQ-score | 45 | 0.84 (0.72-0.94) | 106 | 0.83 (0.68-0.93) | 0.28 |
|  |  | EQ-VAS | 41 | 70 (65-85) | 104 | 70 (60-85) | 0.76 |
|  |  | HADS-Anxiety | 40 | 3.5 (0.5-7) | 96 | 4 (1-8) | 0.36 |
|  |  | HADS-Depression | 40 | 3 (0.5-7.5) | 96 | 2.5 (1-7) | 0.79 |

**Supplemental Table 2: The five components of the EQ-score at Pre-ICU, D90 and Y1 timepoints in the studied population.**

| **Outcomes** | | **Studied population**  **(n=156)** | |
| --- | --- | --- | --- |
|  |  | n | values |
| Mobility | Pre-ICU | 79 | 1 (1-1) |
|  | D90 | 148 | 2 (1-3) |
|  | Y1 | 151 | 2 (1-3) |
| *p value D90 vs pre* | | *<0.0001* | |
| *p value Y1 vs pre* | | *<0.0001* | |
| *P value Y1 vs D90* | | *<0.0001* | |
| Self-care | Pre-ICU | 79 | 1 (1-1) |
|  | D90 | 148 | 1 (1-3) |
|  | Y1 | 151 | 1 (1-2) |
| *p value D90 vs pre* | | *<0.0001* | |
| *p value Y1 vs pre* | | *0.012* | |
| *P value Y1 vs D90* | | *<0.0001* | |
| Usual activities | Pre-ICU | 79 | 1 (1-1) |
|  | D90 | 148 | 2 (1-3) |
|  | Y1 | 151 | 2 (1-3) |
| *p value D90 vs pre* | | *<0.0001* | |
| *p value Y1 vs pre* | | *<0.0001* | |
| *P value Y1 vs D90* | | *<0.0001* | |
| Pain / Discomfort | Pre-ICU | 79 | 1 (1-3) |
|  | D90 | 148 | 2 (1-3) |
|  | Y1 | 151 | 2 (1-3) |
| *p value D90 vs pre* | | *0.011* | |
| *p value Y1 vs pre* | | *0.30* | |
| *P value Y1 vs D90* | | *0.029* | |
| Anxiety / Depression | Pre-ICU | 79 | 1 (1-2) |
|  | D90 | 148 | 1 (1-2) |
|  | Y1 | 151 | 1 (1-2) |
| *p value D90 vs pre* | | *0.33* | |
| *p value Y1 vs pre* | | *0.10* | |
| *P value Y1 vs D90* | | *0.51* | |

*Data are expressed as median (P25-P75)*

*D: day; Y: year*

**Supplemental Table 3: Association between EQ-score evolution and patients clinical and biological characteristics.**

| **Characteristic** | | | **EQ-score Y1 > D90** | | **EQ-score Y1 ≤ D90** | | **p value** |
| --- | --- | --- | --- | --- | --- | --- | --- |
|  |  |  | **n** | **Values** | **n** | **Values** |  |
| Age, years | | | 89 | 60 (50-66) | 55 | 60 (54-68) | 0.32 |
| Sex (female), n (%) | | | 89 | 33 (37.1) | 55 | 22 (40) | 0.73 |
| BMI at D0, kg/m^2^ | | | 79 | 30.5 (26.5 – 35.1) | 51 | 31.2 (26.6-35.9) | 0.61 |
| Educational level, n (%) | Secondary education | | 45 | 26 (57.8) | 37 | 31 (83.8) | 0.047 |
|  | Bachelor’s degree | |  | 8 (17.8) |  | 4 (10.8) |  |
|  | Master’s degree | |  | 5 (11.1) |  | 0 (0) |  |
|  | Doctorate | |  | 3 (6.7) |  | 0 (0) |  |
|  | Other | |  | 3 (6.7) |  | 2 (5.4) |  |
| Employment status, n (%) | Employed | | 62 | 31 (50) | 44 | 18 (40.9) | 0.68 |
|  | Unemployed | |  | 6 (9.7) |  | 3 (6.8) |  |
|  | Retired | |  | 20 (32.2) |  | 20 (45.5) |  |
|  | Sick leave | |  | 4 (6.5) |  | 3 (6.8) |  |
|  | Other | |  | 1 (1.6) |  | 0 (0) |  |
| Clinical Frailty Scale | | | 89 | 3 (2-3) | 55 | 2 (2-3) | 0.58 |
| Comorbidities, n (%) | Diabetes | | 89 | 29 (32.6) | 55 | 13 (23.6) | 0.27 |
|  | Active smoking | | 81 | 7 (8.6) | 50 | 3 (6) | 0.74 |
|  | Active alcohol | | 78 | 2 (2.6) | 54 | 4 (7.4) | 0.22 |
|  | Long-term steroids | | 89 | 13 (14.6) | 55 | 10 (18.2) | 0.57 |
|  | Immunotherapy | | 89 | 6 (6.7) | 55 | 1 (1.8) | 0.25 |
|  | Chemotherapy | | 89 | 1 (1.1) | 55 | 0 (0) | 1 |
| APACHE II | | | 89 | 12 (9-16) | 55 | 12 (8-15) | 0.79 |
| D0 | SOFA | | 89 | 6 (4-7) | 55 | 5 (3-7) | 0.38 |
|  | CRP, mg/L | | 89 | 121 (65-189) | 55 | 107.8 (61-168.3) | 0.47 |
| D7 | SOFA | | 89 | 5 (3-7) | 51 | 4 (3-5) | 0.0013 |
|  | CRP, mg/L | | 89 | 85.4 (31-172.5) | 53 | 63.3(24.1-120.3) | 0.10 |
| Mechanical ventilation duration, days | | | 89 | 20 (12-32) | 54 | 11 (8-21) | 0.0002 |
| Renal replacement therapy, n (%) | | | 89 | 9 (10.1) | 55 | 2 (3.6) | 0.21 |
| ECMO, n (%) | | | 89 | 18 (20.2) | 55 | 4 (7.3) | 0.036 |
| ICU LOS, days | | | 89 | 28 (18-49) | 55 | 11 (8-21) | 0.0002 |
| Hospital LOS, days | | | 89 | 62 (31-87) | 54 | 33 (21-65) | 0.0020 |
| Hospital discharge location, n (%) | | Home | 89 | 54 (60.7) | 55 | 42 (76.4) | 0.18 |
|  |  | Rehabilitation site |  | 28 (31.4) |  | 12 (21.8) |  |
|  |  | Long-term residency |  | 1 (1.1) |  | 0 (0) |  |
|  |  | Other hospital |  | 6 (6.8) |  | 1 (1.8) |  |
|  |  | Other |  | 0 (0) |  | 0 (0) |  |

*Data are expressed as n (%) or median (P25-P50)*

*APACHE: Acute Physiology And Chronic Health Evaluation; BMI: body mass index; D: day; CRP: C-reactive protein; ECMO: Extracorporeal Membrane Oxygenation; EQ: EuroQOL; ICU: intensive care unit; LOS: length of stay; SOFA: Sepsis-related Organ Failure Assessment; VAS: visual analogue scale; Y: year*

**Supplemental Table 4: Association between EQ-VAS evolution and patients clinical and biological characteristics.**

| **Characteristic** | | | **EQ-VAS Y1 > D90** | | **EQ-VAS Y1 ≤ D90** | | **p value** |
| --- | --- | --- | --- | --- | --- | --- | --- |
|  |  |  | **n** | **Values** | **n** | **Values** |  |
| Age, years | | | 77 | 59 (49-68) | 58 | 60.5 (54-65) | 0.40 |
| Sex (female), n (%) | | | 77 | 24 (31.2) | 58 | 28 (48.3) | 0.043 |
| BMI at D0, kg/m^2^ | | | 68 | 30.8 (28.5-34.5) | 54 | 31.2 (26.1-36.1) | 0.97 |
| Educational level, n (%) | Secondary education | | 45 | 31 (68.9) | 37 | 26 (70.3) | 0.97 |
|  | Bachelor’s degree | |  | 7 (15.6) |  | 5 (13.5) |  |
|  | Master’s degree | |  | 3 (6.7) |  | 2 (5.4) |  |
|  | Doctorate | |  | 2 (4.4) |  | 1 (2.7) |  |
|  | Other | |  | 2 (4.4) |  | 3 (8.1) |  |
| Employment status, n (%) | Employed | | 57 | 29 (50.9) | 45 | 18 (40.0) | 0.67 |
|  | Unemployed | |  | 4 (7.0) |  | 5 (11.1) |  |
|  | Retired | |  | 20 (35.1) |  | 18 (40.0) |  |
|  | Sick leave | |  | 3 (5.3) |  | 4 (8.9) |  |
|  | Other | |  | 1 (1.8) |  | 0 (0.0) |  |
| Clinical Frailty Scale | | | 77 | 3 (2-3) | 58 | 3 (2-3) | 0.71 |
| Comorbidities, n (%) | Diabetes | | 77 | 20 (26) | 58 | 19 (32.8) | 0.39 |
|  | Active smoking | | 69 | 6 (8.7) | 53 | 3 (5.7) | 0.73 |
|  | Active alcohol | | 69 | 2 (2.9) | 56 | 4 (7.1) | 0.41 |
|  | Long-term steroids | | 77 | 12 (15.6) | 58 | 11 (19) | 0.60 |
|  | Immunotherapy | | 77 | 5 (6.5) | 58 | 2 (3.4) | 0.70 |
|  | Chemotherapy | | 77 | 1 (1.3) | 58 | 0 (0) | 1 |
| APACHE II | | | 77 | 12 (9-16) | 58 | 12 (9-15) | 0.78 |
| D0 | SOFA | | 77 | 6 (4-7) | 58 | 5 (3-7) | 0.45 |
|  | CRP, mg/L | | 77 | 131.6 (74-189) | 58 | 108.2 (60.7-160) | 0.16 |
| D7 | SOFA | | 76 | 4 (3-6) | 56 | 4 (3-6) | 0.83 |
|  | CRP, mg/L | | 77 | 75.9 (29-154.7) | 57 | 80.8 (20.7-145.9) | 0.57 |
| Mechanical ventilation duration, days | | | 76 | 18.5 (10.5-26.5) | 58 | 12 (9-23) | 0.025 |
| Renal replacement therapy, n (%) | | | 77 | 7 (9.1) | 58 | 4 (6.9) | 0.77 |
| ECMO, n (%) | | | 77 | 14 (18.2) | 58 | 7 (12.1) | 0.33 |
| ICU LOS, days | | | 77 | 27 (17-47) | 58 | 18 (13-35) | 0.0035 |
| Hospital LOS, days | | | 77 | 54 (28-91) | 57 | 41 (24-64) | 0.0026 |
| Hospital discharge location, n (%) | | Home | 77 | 50 (64.9) | 58 | 42 (72.4) | 0.50 |
|  |  | Rehabilitation site |  | 21 (27.3) |  | 15 (25.9) |  |
|  |  | Long-term residency |  | 1 (1.1) |  | 0 (0) |  |
|  |  | Other hospital |  | 5 (6.5) |  | 1(1.7) |  |
|  |  | Other |  | 0 (0) |  | 0 (0) |  |

*Data are expressed as n (%) or median (P25-P50)*

*APACHE: Acute Physiology And Chronic Health Evaluation; BMI: body mass index; D: day; CRP: C-reactive protein; ECMO: Extracorporeal Membrane Oxygenation; ICU: intensive care unit; EQ: EuroQOL; LOS: length of stay; SOFA: Sepsis-related Organ Failure Assessment; VAS: visual analogue scale; Y: year*

**Supplemental Table 5: Duration of mechanical ventilation, ICU stay and hospital stay in the subcohort of 37 patients who did not recover their baseline EQ-score and EQ-VAS ay Y1, according to their evolution between D90 and Y1.**

| **ICU parameters** | **EQ-5D-5L**  **Y1 > D90** | **EQ-5D-5L**  **Y1 ≤ D90** | **p value** |
| --- | --- | --- | --- |
| Mechanical ventilation duration, days | 23 (11-54) | 11 (8.5-21.5) | 0.021 |
| ICU LOS, days | 41 (15-64) | 15 (11-26) | 0.0066 |
| Hospital LOS, days | 84 (28-128) | 26 (19.5-64.5) | 0.0091 |

*Data are expressed as n (%) or median (P25-P50)*

*D: day; ICU: intensive care unit; EQ: EuroQOL; LOS: length of stay; Y: year*
